# Supplementary material for: Validation of the Spanish Versions of FACIT-PAL and FACIT-PAL-14 in Palliative Patients
Source: Int J Environ Res Public Health. 2022 Aug 29;19(17):10731. doi: 10.3390/ijerph191710731 (PMC9518596; doi:10.3390/ijerph191710731)
Supplement: Supplementary file 1 [file ijerph-19-10731-s001.zip › ijerph-1834553-supplementary.pdf]

Final version of FACIT-PAL and FACIT-PAL-14 scales

FACIT-Pal

A continuación, encontrará una lista de declaraciones que otras personas que padecen su misma enfermedad han considerado importantes. Por favor, rodee con un círculo o marque un número por fila para indicar su respuesta referida a los últimos 7 días.

	**BIENESTAR FÍSICO**	**Nada**	**Algo**	**Un poco**	**Bastante**	**Mucho**
GP1	Me siento falto/a de energía	0	1	2	3	4
GP2	Tengo nauseas	0	1	2	3	4
GP3	Mi condición física me impide cubrir las necesidades de mi familia	0	1	2	3	4
GP4	Tengo dolores	0	1	2	3	4
GP5	Estoy preocupado/a por los efectos secundarios del tratamiento	0	1	2	3	4
GP6	Me siento enfermo/a	0	1	2	3	4
GP7	Me veo obligado/a a pasar tiempo en la cama	0	1	2	3	4

	**BIENESTAR SOCIAL/FAMILIAR**	**Nada en absoluto**	**Algo**	**Un poco**	**Bastante**	**Mucho**
GS1	Me siento cercano/a a mis amigos	0	1	2	3	4
GS2	Mi familia me apoya emocionalmente	0	1	2	3	4
GS3	Mis amigos me apoyan	0	1	2	3	4
GS4	Mi familia ha aceptado mi enfermedad	0	1	2	3	4
GS5	Me siento satisfecho/a con la comunicación sobre mi enfermedad	0	1	2	3	4
GS6	Me siento cercano/a a mi pareja (o persona que es mi principal apoyo)	0	1	2	3	4
Q1	*Independientemente de su actual actividad sexual, por favor conteste a la siguiente pregunta. Si prefiere no contestar, por favor marque esta casilla ☐ y pase a la siguiente sección.*					
GS7	Estoy satisfecho/a con mi vida sexual	0	1	2	3	4

FACIT-Pal

Por favor, rodee con un círculo o marque un número por fila para indicar su respuesta referida a los últimos 7 días.

	BIENESTAR EMOCIONAL	Nada	Algo	Un poco	Bastante	Mucho
GE1	Me siento triste...	0	1	2	3	4
GE2	Estoy satisfecho/a con la forma en que estoy llevando mi enfermedad...	0	1	2	3	4
GE3	Estoy perdiendo la esperanza en la lucha contra mi enfermedad.................	0	1	2	3	4
GE4	Estoy nervioso/a..	0	1	2	3	4
GE5	Me preocupa morir	0	1	2	3	4
GE6	Me preocupa que mi condición empeore	0	1	2	3	4

	BIENESTAR FUNCIONAL	Nada	Algo	Un poco	Bastante	Mucho
GF1	Estoy en condiciones de trabajar (incluido el trabajo en casa)	0	1	2	3	4
GF2	Mi trabajo (incluido el trabajo en casa)me resulta enriquecedor...	0	1	2	3	4
GF3	Soy capaz de disfrutar de la vida..	0	1	2	3	4
GF4	He aceptado mi enfermedad... .	0	1	2	3	4
GF5	Duermo bien	0	1	2	3	4
GF6	Estoy disfrutando de las cosas que habitualmente hago por diversión .	0	1	2	3	4
GF7	Estoy contento/a con la calidad de vida que tengo ahora	0	1	2	3	4

FACIT-Pal

Por favor, rodee con un círculo o marque un número por fila para indicar su respuesta referida a los últimos 7 días.

	PREOCUPACIONES ADICIONALES	Nada	Algo	Un poco	Bastante	Mucho
PAL1	Mantengo el contacto con mis amigos.............................	0	1	2	3	4
PAL2	Hay miembros de mi familia que asumirán mis responsabilidades..	0	1	2	3	4
PAL3	Creo que mi familia me aprecia	0	1	2	3	4
PAL4	Siento que soy una carga para mi familia.............................	0	1	2	3	4
B1	Me quedo sin aliento	0	1	2	3	4
PAL5	Estoy estreñido/a	0	1	2	3	4
C2	Estoy perdiendo peso...	0	1	2	3	4
O2	Estoy teniendo vómitos...	0	1	2	3	4
PAL6	Tengo inflamadas partes del cuerpo	0	1	2	3	4
PAL7	Mi boca y garganta están secas..	0	1	2	3	4
Br7	Me siento independiente..	0	1	2	3	4
PAL8	Me siento útil...	0	1	2	3	4
PAL9	Hago que cada día cuente...	0	1	2	3	4
PAL10	Tengo paz mental	0	1	2	3	4
Sp21	Tengo esperanza		1	2	0 3	4
PAL12	Soy capaz de tomar decisiones	0	1	2	3	4
L1	Tengo claridad de pensamiento.....................................	0	1	2	3	4
PAL13	He podido reconciliarme (hacer las paces) con algunas personas...	0	1	2	3	4
PAL14	Puedo comentar abiertamente mis preocupaciones con personas cercanas a mí...	0	1	2	3	4

FACIT-Pal-14

A continuación, encontrará una lista de declaraciones que otras personas que padecen su misma enfermedad han considerado importantes. Por favor, rodee con un círculo o marque un número por fila para indicar su respuesta referida <u>a los últimos 7 días.</u>

		Nada	Algo	Un poco	Bastante	Mucho
GP1	Me siento falto/a de energía	0	1	2	3	4
GP2	Tengo nauseas	0	1	2	3	4
GP4	Tengo dolores	0	1	2	3	4
GE6	Me preocupa que mi condición empeore	0	1	2	3	4
GF3	Soy capaz de disfrutar de la vida	0	1	2	3	4
GF5	Duermo bien	0	1	2	3	4
GF7	Estoy contento/a con la calidad de vida que tengo ahora	0	1	2	3	4
GS2	Mi familia me apoya emocionalmente	0	1	2	3	4
Sp21	Tengo esperanza	0	1	2	3	4
GE1	Me siento triste	0	1	2	3	4
Pal4	Siento que soy una carga para mi familia	0	1	2	3	4
Pal5	Estoy estreñido/a	0	1	2	3	4
Pal14	Puedo comentar abiertamente mis preocupaciones con personas cercanas a mí	0	1	2	3	4
B1	Me quedo sin aliento	0	1	2	3	4
